# Supplementary material for: Socioeconomic Health Inequalities in Adolescent Metabolic Syndrome and Depression: No Mediation by Parental Depression and Parenting Style
Source: Int J Environ Res Public Health. 2021 Jul 20;18(14):7716. doi: 10.3390/ijerph18147716 (PMC8303316; doi:10.3390/ijerph18147716)
Supplement: Supplementary file 1 [file ijerph-18-07716-s001.zip › Supplementary document S1.pdf]

Supplementary document 1

The following R code was used to construct our continuous metabolic syndrome scores. Our methods are based on the approach described by Eisenmann et al. [20].

```
# Variables ending in 1a are from the baseline assessment,
# and variables ending in 2a are from the second assessment.

# Creating the baseline cMetS scores
# First regressing the variables onto age and sex, then standardizing them, and finally
# sum up the individual components.

fasting.glu1a.lm <- lm(fasting_glu_1a ~ age1a + sex, na.action = na.exclude, data = mydata)
mydata$fasting_glu1a_stdres <- as.numeric(scale(residuals(fasting.glu1a.lm)))

wc1a.lm <- lm(waist_c_1a ~ age1a + sex, na.action = na.exclude, data = mydata)
mydata$wc1a_stdres <- as.numeric(scale(residuals(wc1a.lm)))

map1a.lm <- lm(map_1a ~ age1a + sex, na.action = na.exclude, data = mydata)
mydata$map1a_stdres <- as.numeric(scale(residuals(map1a.lm)))

hdc1a.lm <- lm(hdc_1a ~ age1a + sex, na.action = na.exclude, data = mydata)
mydata$hdc1a_stdres <- as.numeric(scale(residuals(hdc1a.lm))) * -1

tgl1a.lm <- lm(tgl_1a ~ age1a + sex, na.action = na.exclude, data = mydata)
mydata$tgl1a_stdres <- as.numeric(scale(residuals(tgl1a.lm)))

mydata$cmets1a <- with(mydata, fasting_glu1a_stdres + wc1a_stdres + map1a_stdres +
hdc1a_stdres + tgl1a_stdres)


# Creating the second assessment cMetS scores
# First standardizing the individual components of MetS using the regression coefficients
# and residual standard deviations from the baseline assessment. Lastly, adding the
# components together. Males are the reference group in the regressions, so (sex ==
# "Female") ensures that the beta is only added for females. When the individual is a male
# the logical statement is false, and this becomes a 0 in the calculation.

res_glu <- summary(fasting.glu1a.lm)$coefficients[,"Estimate"]
mydata$fasting_glu2a_stdres <- with(mydata, (fasting_glu_2a - (res_glu[1] + age2a *
res_glu[2] + (sex=="Female") * res_glu[3])) / sd(residuals(fasting.glu1a.lm), na.rm = T))

res_wc <- summary(wc1a.lm)$coefficients[,"Estimate"]
mydata$wc2a_stdres <- with(mydata, (waist_c_2a - (res_wc[1] + age2a * res_wc[2] +
(sex=="Female") * res_wc[3])) / sd(residuals(wc1a.lm), na.rm = T))

res_map <- summary(map1a.lm)$coefficients[,"Estimate"]
```

```
mydata$map2a_stdres <- with(mydata, (map_2a - (res_map[1] + age2a * res_map[2] +
(sex=="Female") * res_map[3])) / sd(residuals(map1a.lm), na.rm = T) )

res_hdc <- summary(hdc1a.lm)$coefficients[,"Estimate"]
mydata$hdc2a_stdres <- with(mydata, (hdc_2a - (res_hdc[1] + age2a * res_hdc[2] +
(sex=="Female") * res_hdc[3])) / sd(residuals(hdc1a.lm), na.rm = T) * -1)

res_tgl <- summary(tgl1a.lm)$coefficients[,"Estimate"]
mydata$tgl2a_stdres <- with(mydata, (tgl_2a - (res_tgl[1] + age2a * res_tgl[2] +
(sex=="Female") * res_tgl[3])) / sd(residuals(tgl1a.lm), na.rm = T))

mydata$cmets2a <- with(mydata, fasting_glu2a_stdres + wc2a_stdres + map2a_stdres +
hdc2a_stdres + tgl2a_stdres)


# Creating cMetS change score by subtracting the baseline values from the second
# assessment values

mydata$cmetsdif <- mydata$cmets2a - mydata$cmets1a
```
