# Supplementary figures and images for: Socioeconomic Health Inequalities in Adolescent Metabolic Syndrome and Depression: No Mediation by Parental Depression and Parenting Style
Source: Int J Environ Res Public Health. 2021 Jul 20;18(14):7716. doi: 10.3390/ijerph18147716 (PMC8303316; doi:10.3390/ijerph18147716)

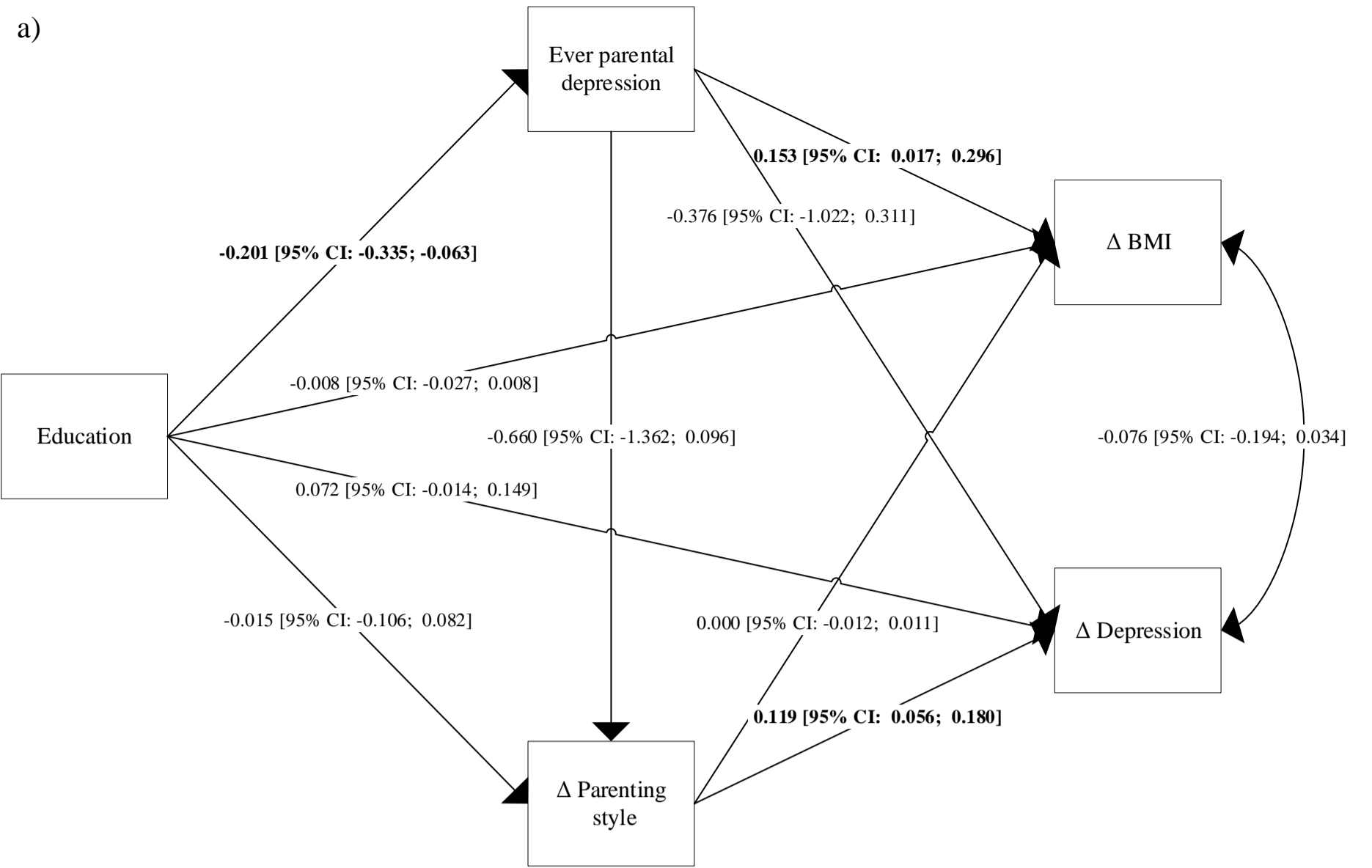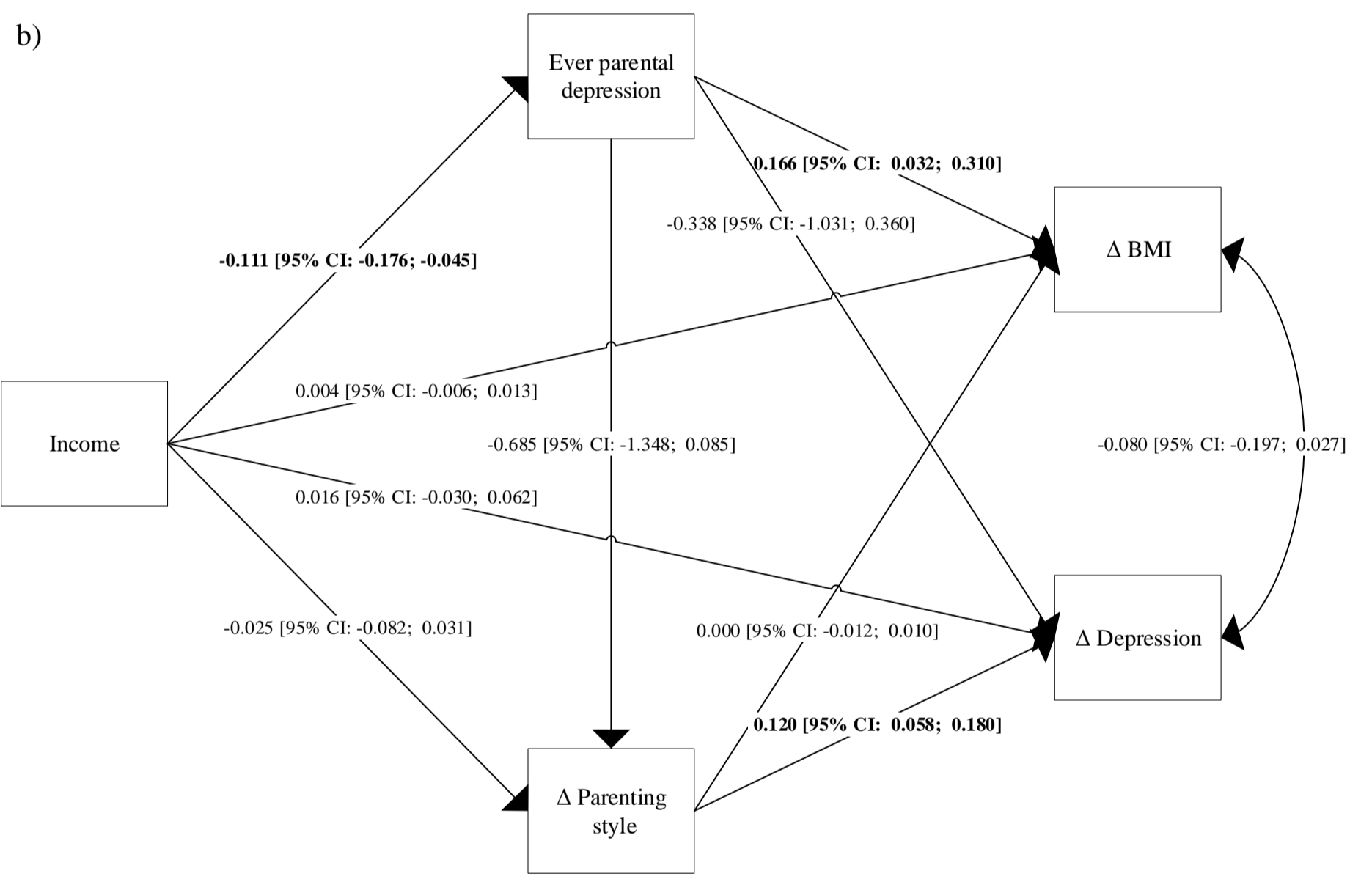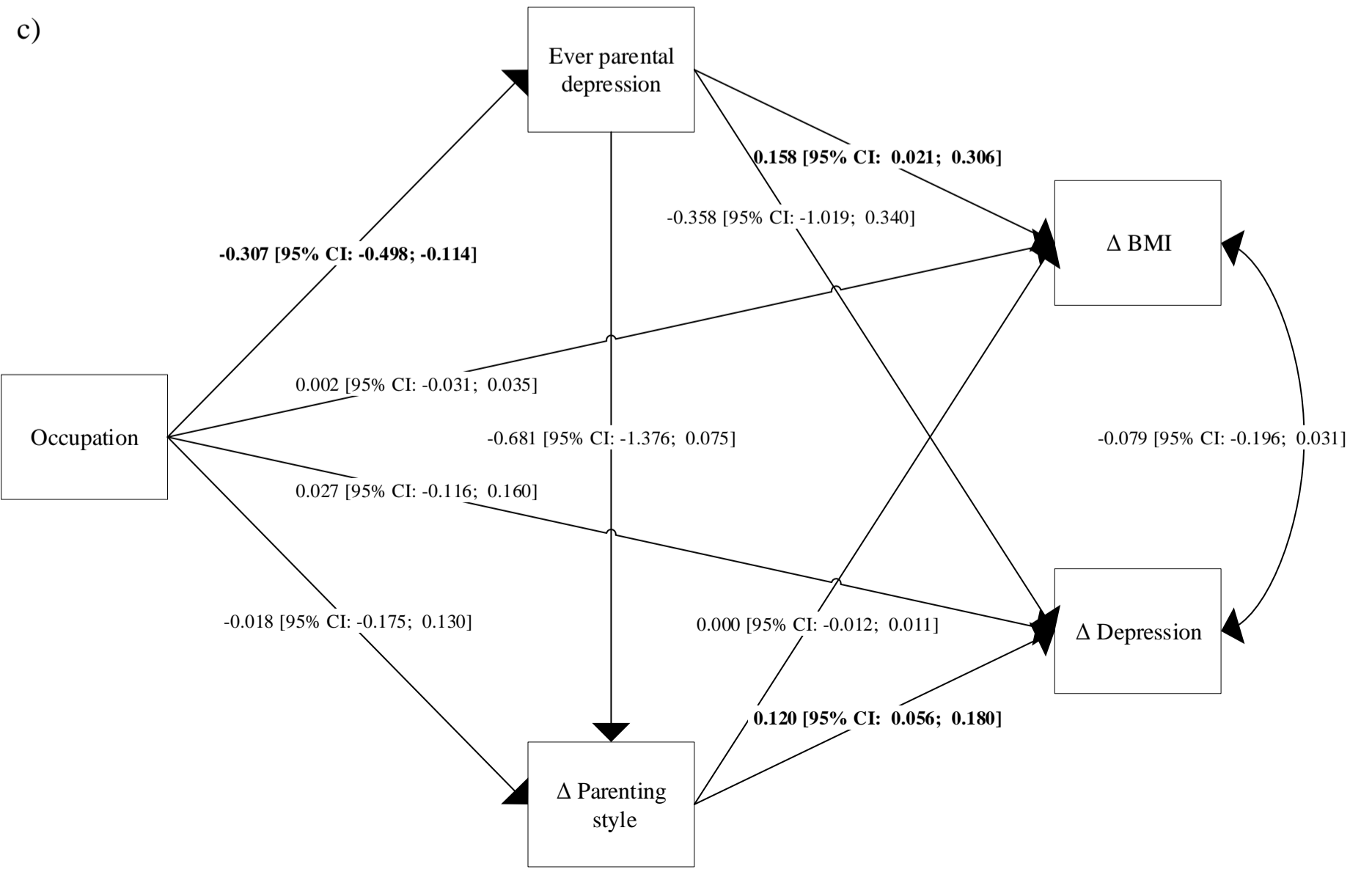

Supplement: Supplementary file 1 [file ijerph-18-07716-s001.zip › Figure S3.pdf]

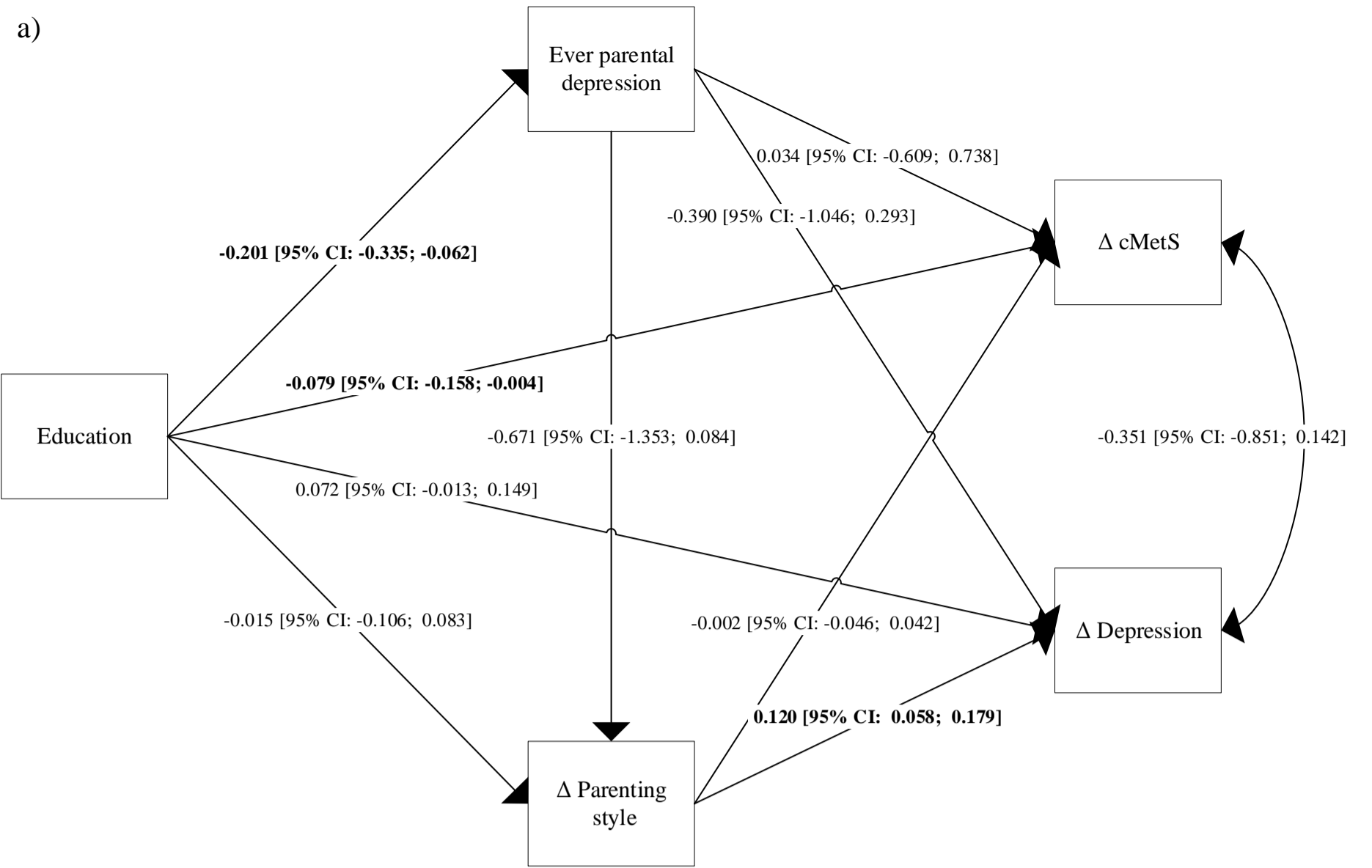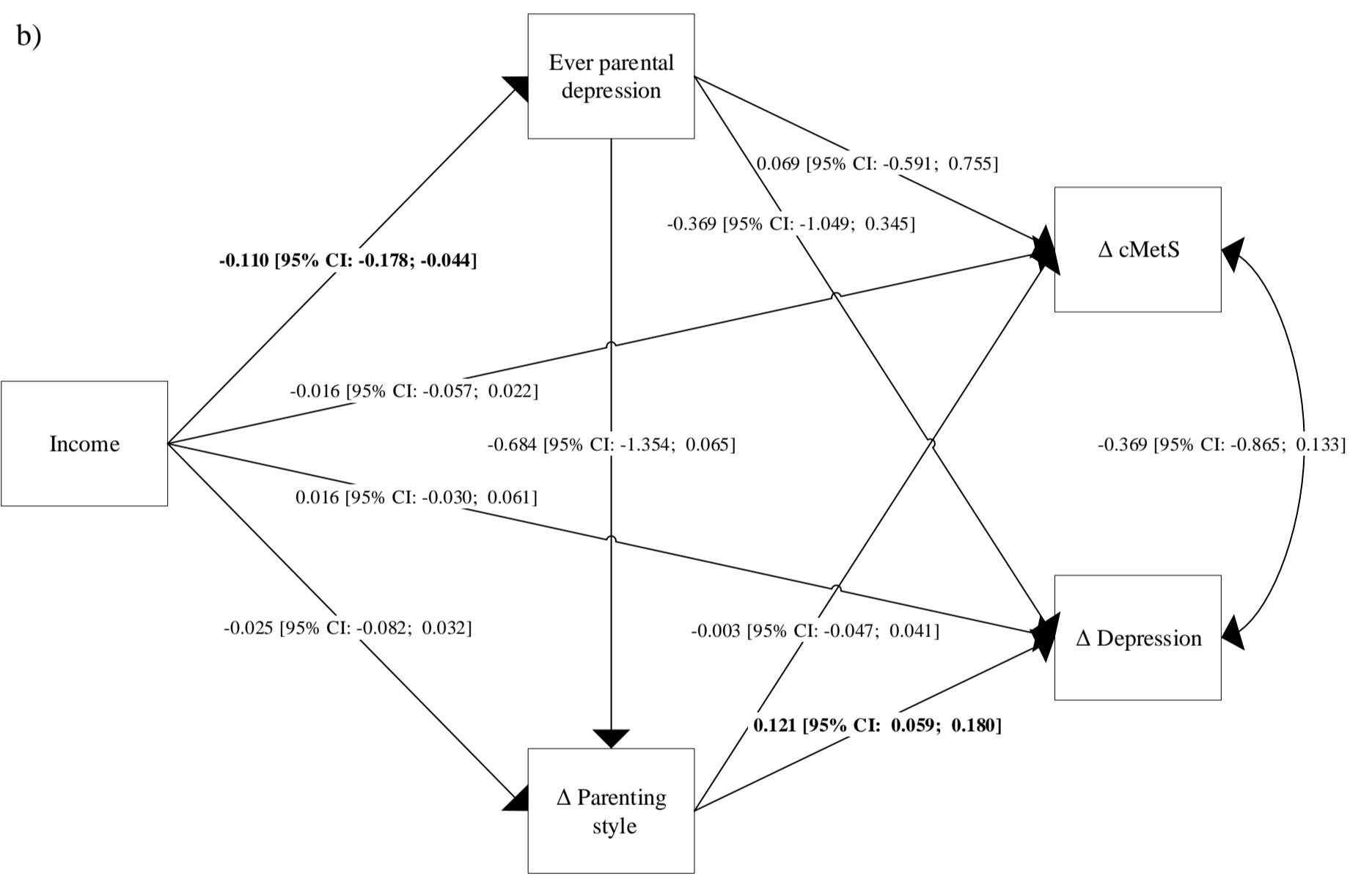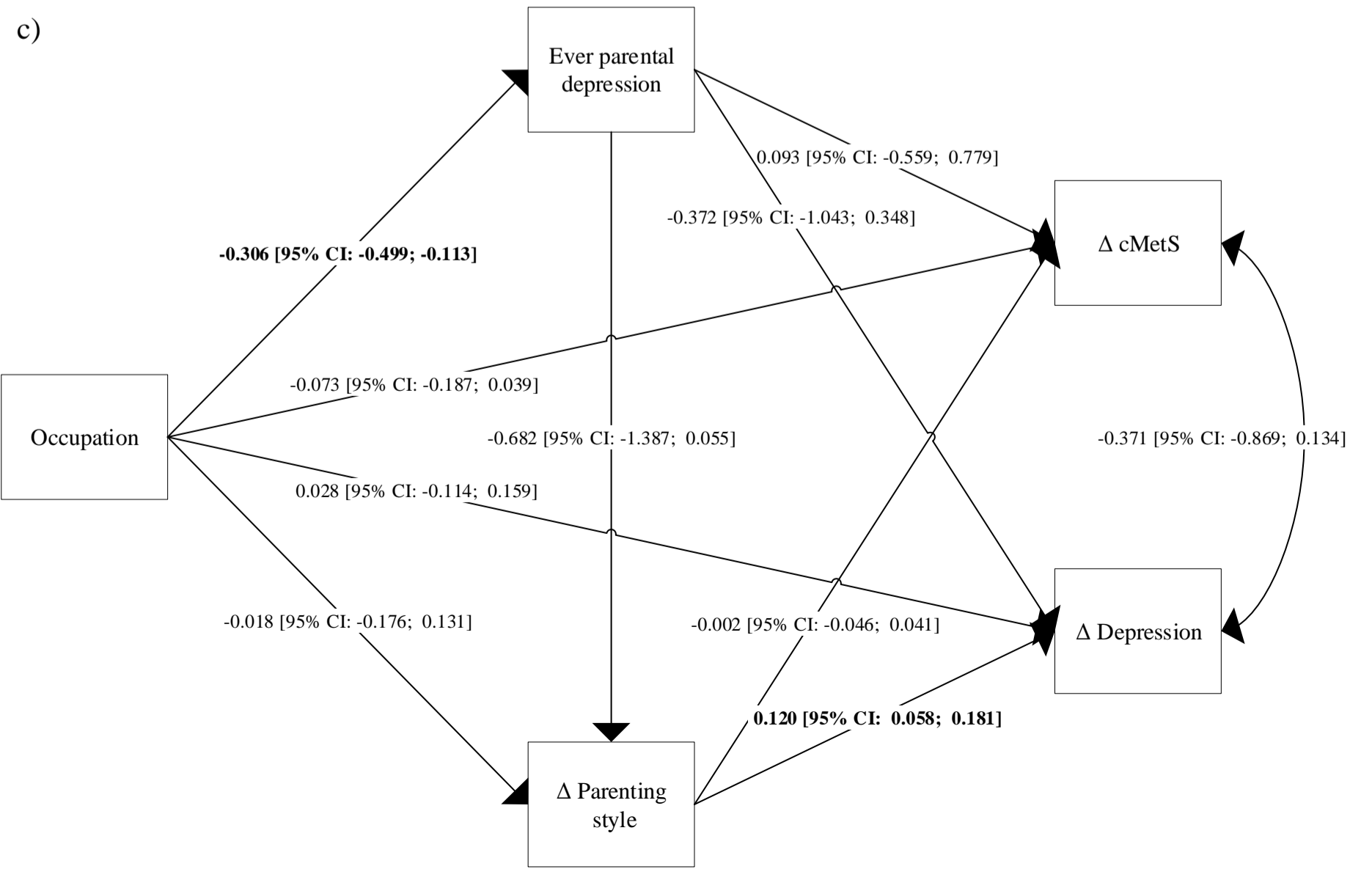

Supplement: Supplementary file 1 [file ijerph-18-07716-s001.zip › Figure S4.pdf]

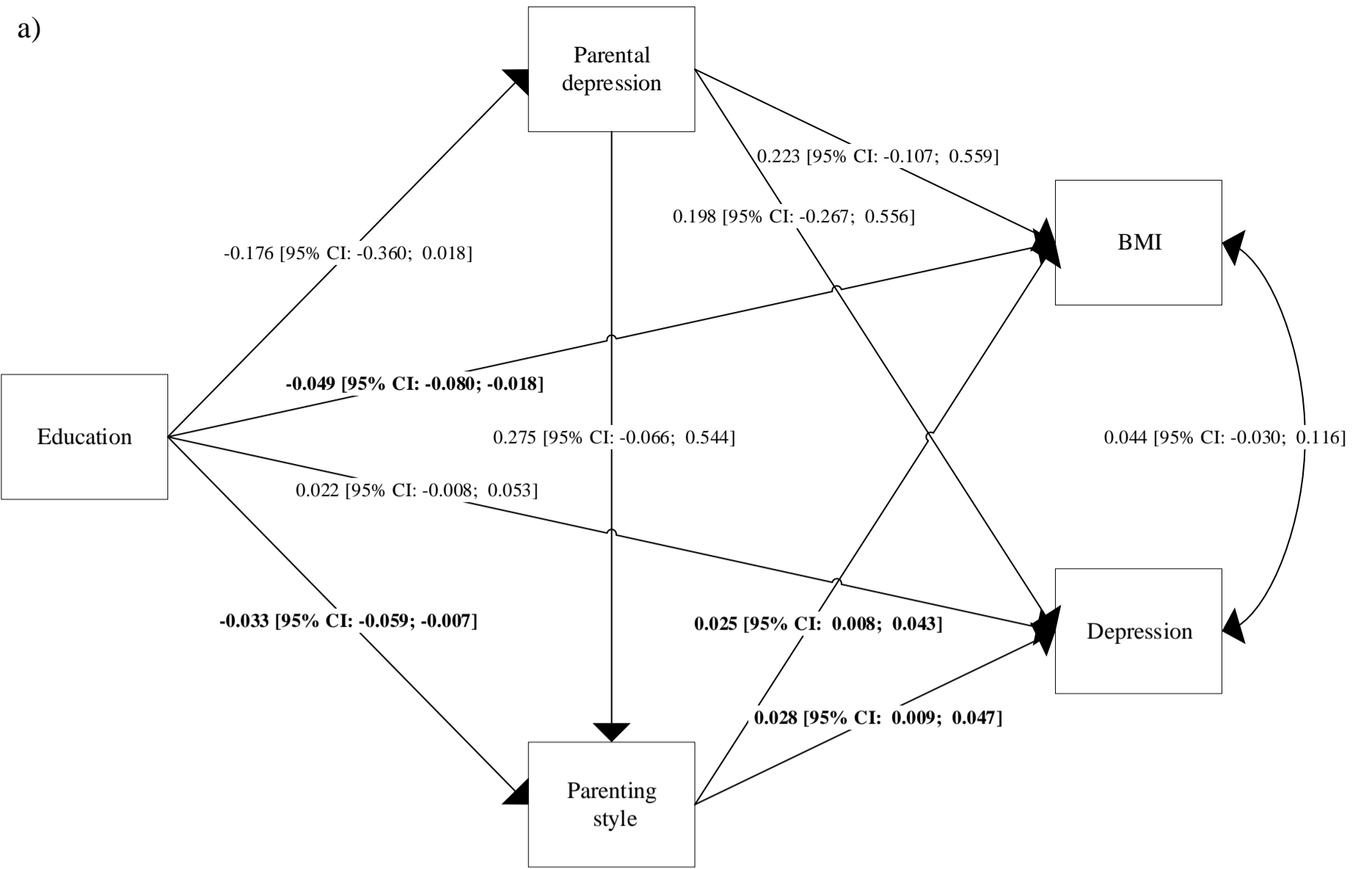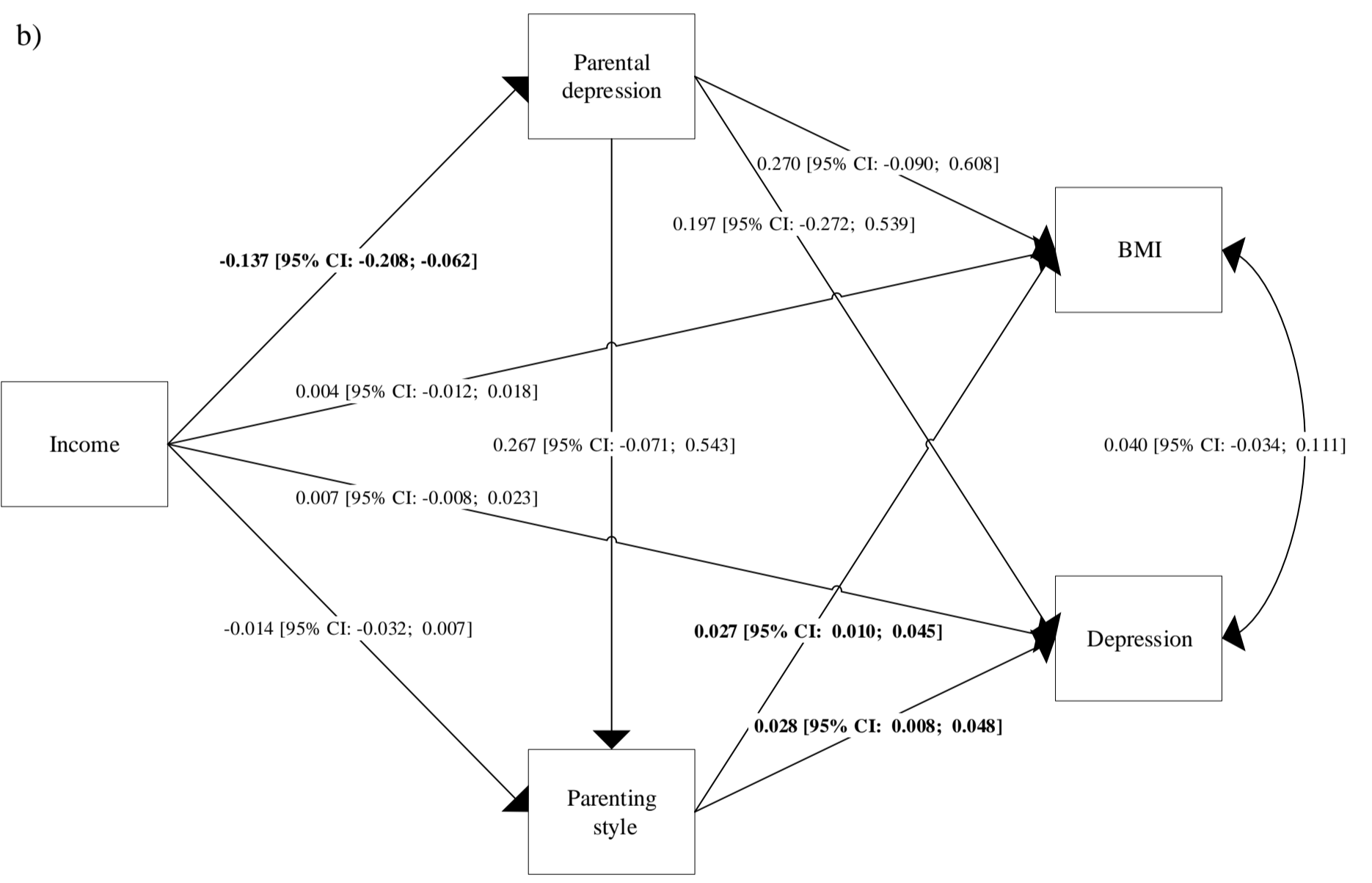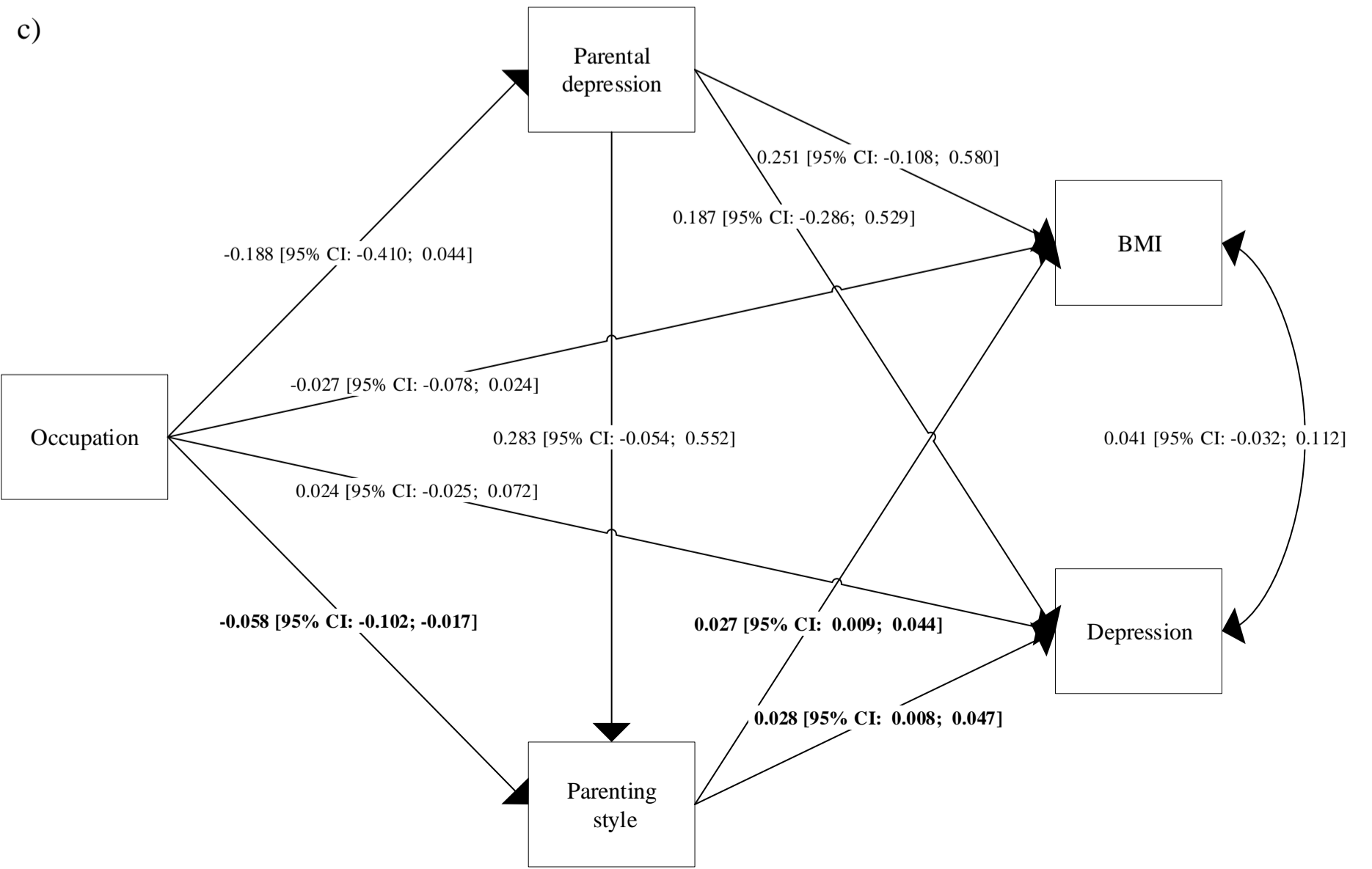

Supplement: Supplementary file 1 [file ijerph-18-07716-s001.zip › Figure S1.pdf]

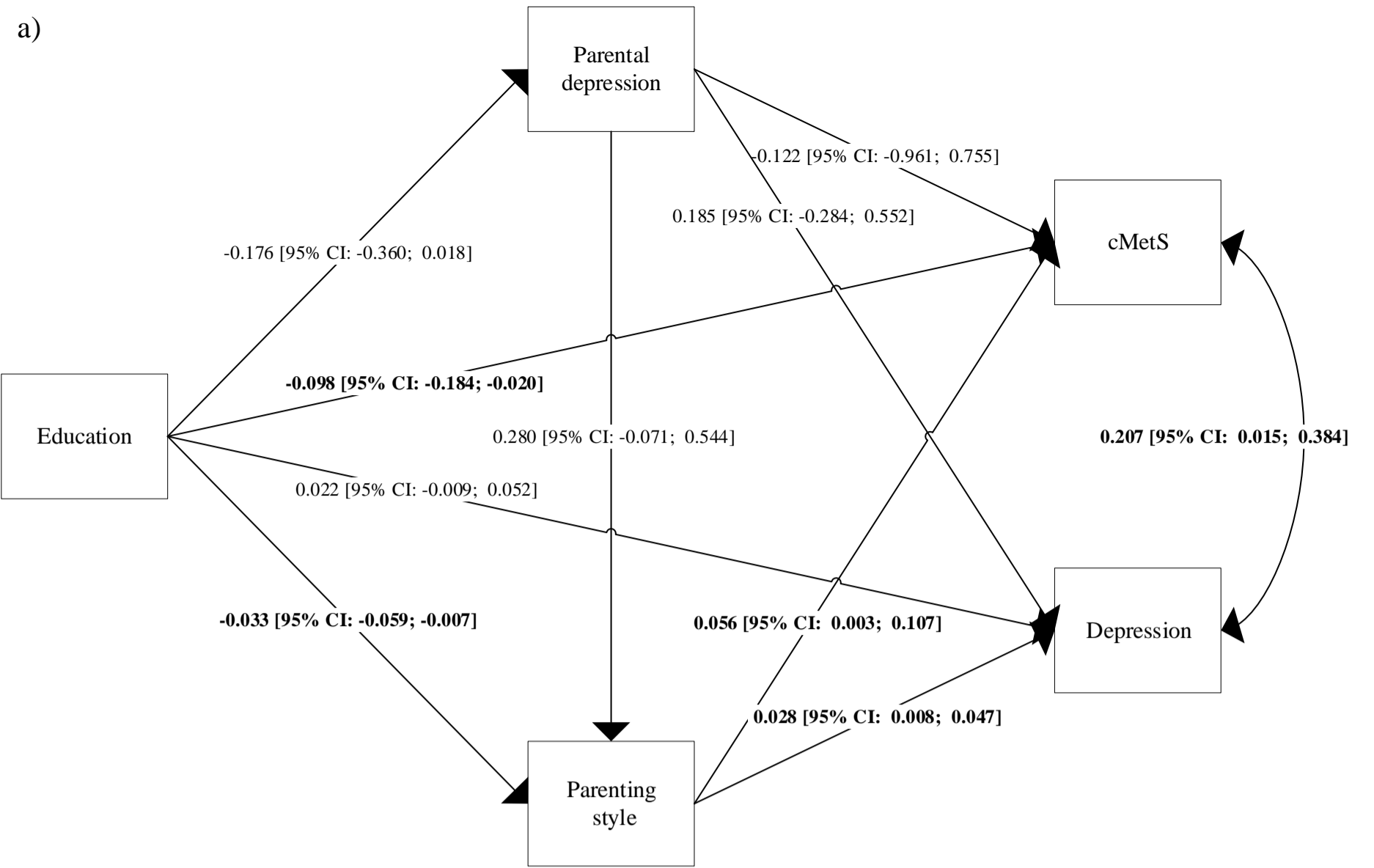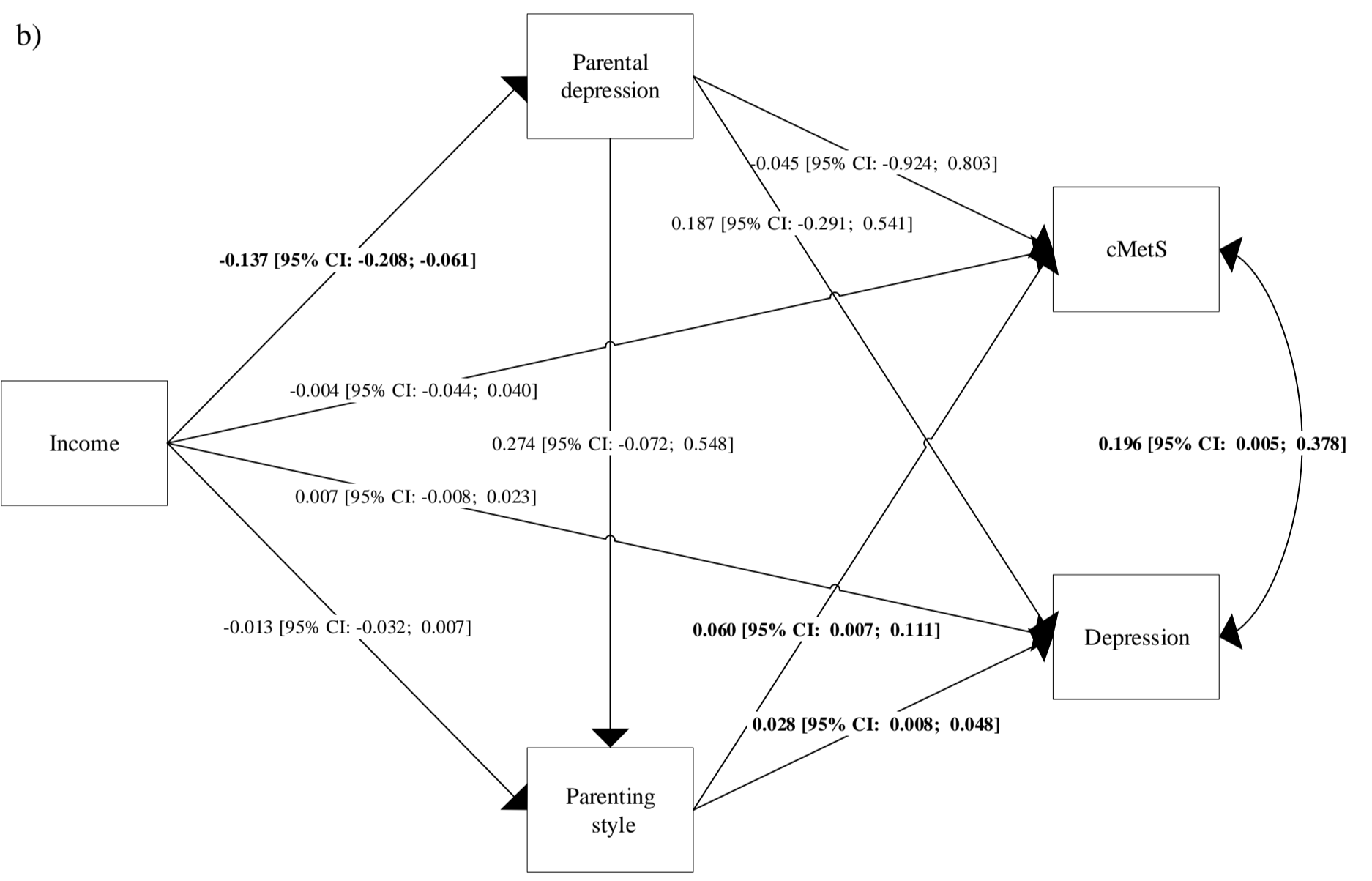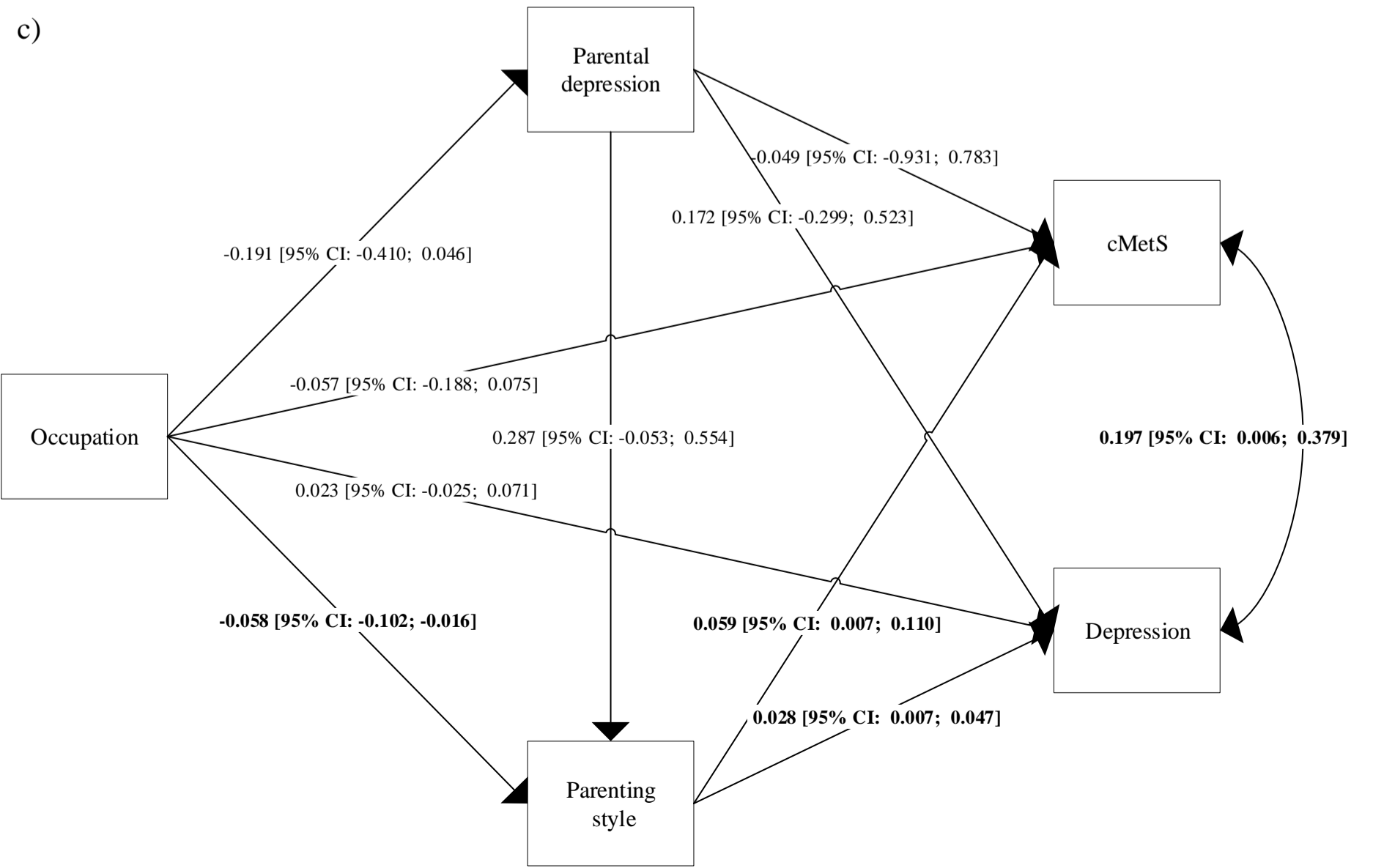

Supplement: Supplementary file 1 [file ijerph-18-07716-s001.zip › Figure S2.pdf]
